# Cuticle Structure in Relation to Chemical Composition: Re-assessing the Prevailing Model

**DOI:** 10.3389/fpls.2016.00427

**Published:** 2016-03-31

**Authors:** Victoria Fernández, Paula Guzmán-Delgado, José Graça, Sara Santos, Luis Gil

**Affiliations:** ^1^Forest Genetics and Ecophysiology Research Group, Plant Physiology and Anatomy Unit, School of Forest Engineering, Technical University of MadridMadrid, Spain; ^2^Department of Plant Sciences, University of California, Davis, DavisCA, USA; ^3^Centro de Estudos Florestais, Instituto Superior de Agronomia, Universidade de LisboaLisboa, Portugal

**Keywords:** cuticle, cell wall, cutin, epidermis, plant surfaces, polysaccharides, waxes

## Abstract

The surface of most aerial plant organs is covered with a cuticle that provides protection against multiple stress factors including dehydration. Interest on the nature of this external layer dates back to the beginning of the 19th century and since then, several studies facilitated a better understanding of cuticular chemical composition and structure. The prevailing undertanding of the cuticle as a lipidic, hydrophobic layer which is independent from the epidermal cell wall underneath stems from the concept developed by Brongniart and von Mohl during the first half of the 19th century. Such early investigations on plant cuticles attempted to link chemical composition and structure with the existing technologies, and have not been directly challenged for decades. Beginning with a historical overview about the development of cuticular studies, this review is aimed at critically assessing the information available on cuticle chemical composition and structure, considering studies performed with cuticles and isolated cuticular chemical components. The concept of the cuticle as a lipid layer independent from the cell wall is subsequently challenged, based on the existing literature, and on new findings pointing toward the cell wall nature of this layer, also providing examples of different leaf cuticle structures. Finally, the need for a re-assessment of the chemical and structural nature of the plant cuticle is highlighted, considering its cell wall nature and variability among organs, species, developmental stages, and biotic and abiotic factors during plant growth.

## Introduction

Cuticles are the interface between non-woody aerial plant organs and the surrounding atmosphere (Riederer and Schreiber, 2001). In general, the cuticle is located at the external, periclinal cell wall of epidermal cells, being also projected between anticlinal walls (Javelle et al., 2011) and sometimes covering the cell walls bordering substomatal chambers (Osborn and Taylor, 1990). It extends for example, over leaf (e.g., Bessire et al., 2007; Kosma et al., 2010; Buschhaus and Jetter, 2012), flower petal (Li-Beisson et al., 2009; Panikashvili et al., 2011; Mazurek et al., 2013; Buschhaus et al., 2015), primary stem (Xue et al., 2014), fruit (Khanal et al., 2011; Lara et al., 2014, 2015; Martin and Rose, 2014) and trichome (Fernández et al., 2011, 2014b) surfaces. A protective role of plant cuticles has been recognized in relation to, for instance, limiting water loss (Kerstiens, 1996; Riederer and Schreiber, 2001), pathogen (Serrano et al., 2014) and insect attack (Eigenbrode and Jetter, 2002), or attenuating UV irradiation (Krauss et al., 1997).

The multi-functional character of the cuticle is achieved by a heterogeneous structural and chemical nature (Khayet and Fernández, 2012) which may additionally vary between e.g., species, genotypes, organs, developmental stages, plant physiological status, or environmental conditions during growth (e.g., Knoche et al., 2004; Szakiel et al., 2012; Nawrath et al., 2013; Fernández et al., 2014a; Guzmán-Delgado et al., 2016).

**FIGURE 1:**
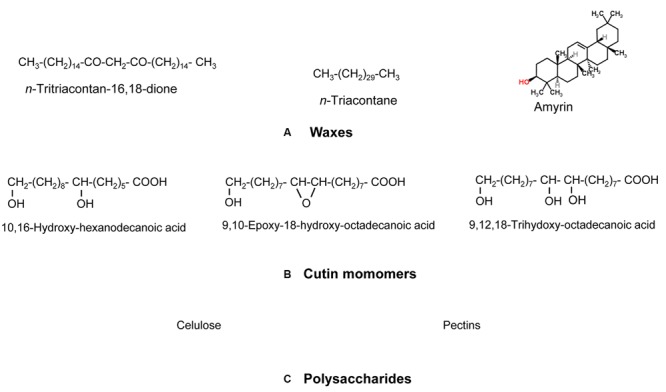
**Model chemical compounds commonly found in plant cuticles, organized according to a decreasing gradient of apolarity: **(A)** Waxes (predominantly apolar), **(B)** cutin monomers (with a large apolar component but having some degree of polarity and hydrogen (H)-bonding interactions due to the presence of functional groups containing oxygen (O)) and **(C)** Polysaccharides (with a lower apolar component but higher polarity and very high H-bonding interactions due to the presence of functional groups with O).** Modified from Khayet and Fernández (2012).

From a chemical viewpoint, the cuticle is formed by an array of compounds with different physico-chemical properties (see **Figure 1** as an example of common cuticle constituents). These compounds can be waxes, cutin and/or cutan, polysaccharides, phenolics and mineral elements (España et al., 2014; Guzmán et al., 2014a; Guzmán-Delgado et al., 2016). Cuticular waxes are a mixture of compounds, such as long-chain fatty acids, alcohols, alkanes, esters or triterpenoids (Jetter et al., 2006). Cutin is defined as a polyester mainly formed by C_16_ and C_18_ hydroxy and hydroxy-epoxy fatty acid monomers (Kolattukudy, 1970), while other compounds such as glycerol have also been suggested to be part of the cutin polymer (Graça et al., 2002). The chemical nature of cutan, an alternative highly insoluble and non-deesterifiable compound found in the cuticle of many species and organs (e.g., Boom et al., 2005; Johnson et al., 2007) is still unclear. Cutan may be formed by polymethylenic and polysaccharide moieties linked via non-hydrolyzable bonds (Nip et al., 1986; Tegelaar et al., 1991), or exclusively by a network of polymethylenic chains containing double bonds and free carboxylic groups linked by ether bonds (Villena et al., 1999). Other researchers proposed that the structure of cutan may be based on aromatic domains with additional carboxylic functional groups ester-linked to long-chain alcohols and long-chain carboxylic acids, respectively (McKinney et al., 1996; Deshmukh et al., 2005).

**FIGURE 2:**
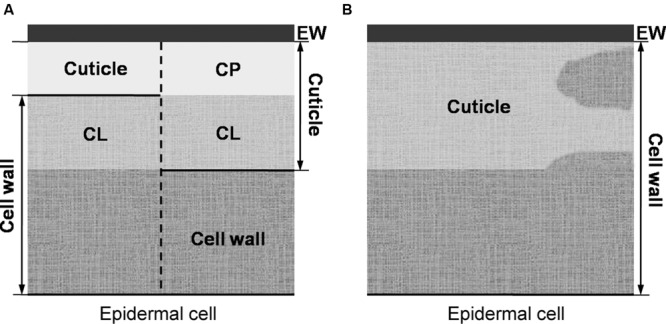
**(A**, left side) Preliminary model of von Mohl (1847) in which the cuticle is restricted to its outermost cellulose-free region (i.e., the cuticle proper), as basis for (**A**, right side) the prevailing plant cuticle model in which the cuticle proper is believed to be free of polysaccharides. **(B)** The cuticle understood as a lipidized, chemically and structurally heterogeneous region of the epidermal cell wall.

The prevailing model (as described in, e.g., Evert, 2006 or Albersheim et al., 2011) considers the cuticle as a lipidic layer whose relationship with the cell wall is restricted to their adjacent position (see **Figure 2A**). According to this model, a cutin matrix with embedded intracuticular waxes and phenolics extends through the cuticle, while polysaccharides are restricted to the innermost cuticle region, i.e., that in contact with the cell wall underneath (Domínguez et al., 2011). An additional layer of epicuticular waxes is deposited on to the cutin matrix and constitutes the organ-atmosphere interface (Domínguez et al., 2011).

In recent decades, substantial progress has been made for characterizing the mechanisms of wax synthesis and export (Samuels et al., 2008; Kunst and Samuels, 2009), cutin monomer synthesis and assembly (e.g., Li-Beisson et al., 2009; Domínguez et al., 2010; McFarlane et al., 2010; Girard et al., 2012; Yeats et al., 2012), and structural and chemical responses of the cuticle to biotic and abiotic stress factors (e.g., Bessire et al., 2007; Isaacson et al., 2009; Kosma et al., 2009), also in relation to different stages of organ development (e.g., España et al., 2014). However, many aspects of cuticle structure in relation to chemical composition (Guzmán et al., 2014a; Guzmán-Delgado et al., 2016) remain unclear e.g., cuticle genesis, or cuticular component synthesis and transport (Bird, 2008; Pollard et al., 2008; Khayet and Fernández, 2012; Domínguez et al., 2015).

For improving the current state of knowledge on the nature of the plant cuticle it is key to recognize that much effort has been devoted to characterize the plant epidermis and also the plant cuticle since the 17th century. While some authors (e.g., Brongniart, 1830; von Mohl, 1847) believed that the cuticle was a distinct layer from the epidermal cell wall, an idea that still prevails nowadays, this concept was already controversial for some researchers of the 19th century (e.g., Meyen, 1837; Fremy et Terreil, 1868). Thereby, with the aim of bringing again into debate the nature of the plant cuticle understood as an independent and lipidic layer deposited on to the epidermal cell wall versus a part of the epidermal cell wall in itself, a historical overview of plant cuticle studies is first provided.

## The Plant Cuticle: A Rancid Research Topic

The term ‘cuticle’ was introduced in the field of plants by Grew (1672) and Malpighi (1675) for referring to the external part of the their organs. The concept of the cuticle as a distinct layer from the epidermis was simultaneously introduced by Brongniart (1830) and Henslow (1831). Both authors described the cuticle as a fine, homogeneous and continuous ‘film’ (Brongniart, 1830) or ‘membrane’ (Henslow, 1831) that covered the epidermal cells.

Treviranus (1835) confirmed the existence of the cuticle and considered it a continued deposition of ‘coagulable matter.’ Meyen (1837) regarded the cuticle as the thickened outer wall of the epidermal cells. Schleiden (1842) considered the cuticle as a ‘mass’ secreted by epidermal cells which subsequently hardens and forms a network.

The occurrence of a layered cuticle was introduced by von Mohl (1842, 1847), giving rise to the morphological model that prevails to date (**Figure 2A**). von Mohl (1847) stressed that the cuticle should be distinguished from the subjacent epidermal cells. This author restricted the term ‘cuticle’ to the outermost, cellulose-free region (as depicted in **Figure 2A**). The region underneath, was referred to as ‘cuticular layer (of the cell wall).’ The name ‘epicuticular waxes’ is nowadays used to distinguish the outermost chemical compounds from those occurring in internal cuticle regions (i.e., the ‘intracuticular waxes’; Jetter and Riederer, 2016).

During the 20th century, additional terms were also suggested for the regions referred by von Mohl to as ‘cuticle’ and ‘cuticular layer.’ The widespread use of terms related to ‘cutin’ (for instance, ‘cutinized’) when referring to the cuticle and its parts provide evidence for the major role attributed to this polyester (e.g., Riederer and Schönherr, 1988; Franke et al., 2005; Pollard et al., 2008; Domínguez et al., 2015). In various reports, terms such as ‘cutin layer’ or ‘cutinized (cell) wall’ have often been employed as synonymous of ‘cuticle’ (e.g., Grzegorzewski et al., 2010; Domínguez et al., 2011; Krajšek et al., 2011; Bellow et al., 2012; Petit et al., 2014). In addition, some authors considered cutin as the structural component of the cuticle (Kolattukudy, 1970, 2005).

When analyzing the leaf cuticle of pear (*Pyrus communis*), Norris and Bukovac (1968) used the term cuticle to ‘include all the layers that can be separated from the underlying cellulose cell wall.’ However, many plant cuticles from different species and organs cannot be isolated from the underlying tissues as intact layers of significant size (e.g., Gouret et al., 1993; Fernández et al., 2014a; Guzmán et al., 2014b).

From this brief historical overview it can be concluded that since approximately two centuries several researchers attempted to characterize the chemical and structural nature of plant cuticles and cuticular layers, giving rise to multiple concepts and names such as ‘cuticle,’ ‘cuticular layer,’ ‘cuticle proper’ or ‘epicuticular waxes.’ However, it is also clear that many controversies remain open such us using the concept of the cuticle *sensu* Norris and Bukovac (1968; i.e., including all the layers that can be separated from the underlying cellulose cell wall), or the definition of a ‘cuticle proper’ free from polysaccharides as highlighted by (Guzmán et al., 2014a,c).

## Cuticular Ultra-Structure in Relation to Chemical Composition: A Difficult and Variable Relationship

The number of studies directly devoted to examine the relationship between internal cuticle structure and chemical composition are scarce (e.g., Wattendorff and Holloway, 1982; Riederer and Schönherr, 1988; Gouret et al., 1993; Viougeas et al., 1995; Krüger et al., 1996; Domínguez and Heredia, 1999a; Graça and Lamosa, 2010; Guzmán et al., 2014a; Guzmán-Delgado et al., 2016). In addition, a high proportion of the investigations performed during the last four decades focused on the cuticle of *Agave americana* (e.g., Wattendorff and Holloway, 1982; Villena et al., 1999; Reina et al., 2007) and *Clivia miniata* leaves (e.g., Schmidt and Schönherr, 1982; Domínguez and Heredia, 1999a; Fagerström et al., 2013), or tomato fruit (*Lycopersicon esculentum*; e.g., Petracek and Bukovac, 1995; Benítez et al., 2004; López-Casado et al., 2007; Segado et al., 2016).

**FIGURE 3:**
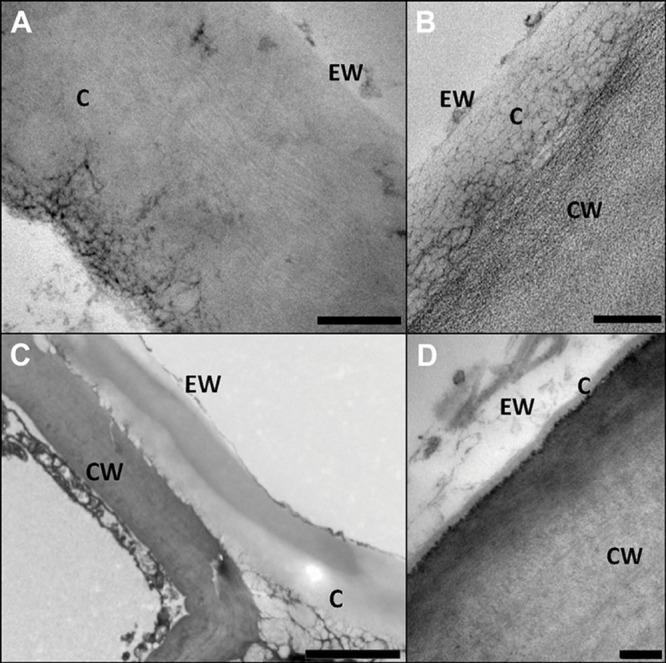
**Structural heterogeneity of plant cuticles, exemplified by transversal TEM leaf cuticle sections of: **(A)** pear (*Pyrus communis*; bar, 200 nm), **(B)** poplar (*Populus bolleana;* bar, 200 nm), **(C)** Magellan’s beech (*Nothofagus betuloides*; bar, 2 μm), and **(D)** wheat (*Triticum aestivum*; bar, 50 nm).** Letters indicate areas corresponding to epicuticular waxes (EW), cuticle (C) and cell wall (CW). Micrographs by V. Fernández and P. Guzmán (2012, 2015).

Transmission electron microscopy (TEM) was especially employed during the early 1980’s for characterizing internal cuticle ultra-structure (Holloway, 1982; Jeffree, 2006). According to the appearance of cuticle internal regions, Holloway (1982) suggested a morphological classification including six cuticular types. Nevertheless, the author highlighted the heterogeneity in plant cuticle structure and the need to consider each species individually to avoid oversimplifications and generalizations. Moreover, structural differences can be observed within the same organ, species and even within a cuticle section analyzed by TEM, hence making it risky to establish broad conclusions (**Figure 3C**; Guzmán et al., 2014b). In this regard, the assignment of the cuticle of a number of species to one or other morphological type may vary, for example, according to the interpretation of the authors (Jeffree, 2006), or to the sample preparation procedure used for TEM observation (Guzmán et al., 2014b).

In **Figure 3** the ultra-structure of the leaf cuticle of three different plant species is shown as an example of the structural heterogeneity of plant cuticles. The pear leaf cuticle is much thicker (∼800 nm) than those of poplar (*Populus bolleana*, ∼300 nm) and chiefly wheat (*Triticum aestivum*) leaf (∼40 nm). This implies a predominance of epicuticular waxes and a tinny cuticle development in some wheat leaf areas (**Figure 3D**; Fernández et al., 2014a), versus a thicker cuticle development in deciduous poplar and pear leaves (Guzmán et al., 2014c). By contrast, the Magellan’s beech (*Nothofagus betuloides*) leaf examined had irregular and large electron-dense areas in the cuticle, similar to those observed in *Ficus elastica* (Guzmán-Delgado et al., 2016).

The complex and composite physical and chemical nature of the cuticle makes it difficult to ascertain the localization of individual constituents and fractions in cuticle transversal sections (Guzmán et al., 2014a,b; Guzmán-Delgado et al., 2016). In addition, the molecular self-assembly and the potential linkage mechanisms among cuticle constituents are little understood (Pollard et al., 2008; Kunst and Samuels, 2009). Several factors may influence cuticle transversal sections as observed by TEM and also by optical-based techniques. These techniques have been also used in combination with histochemical stains and/or chemical extractions to gain information about cuticle morphology and gross chemical composition (e.g., Roberts et al., 1948; Norris and Bukovac, 1968; López-Casado et al., 2007; Buda et al., 2009). For example, the different reactants used for sample preparation (e.g., organic solvents, resins, or stains) may not easily infiltrate cuticular nano-pores. This may be even more difficult for chemicals dissolved in water, a liquid having a high surface tension as discussed by Guzmán et al. (2014b). Therefore, it is likely that the zones which are more superficial and accessible for infiltration in chemical and physical terms will be better fixed, contrasted and distinguished (Guzmán et al., 2014b). Thus, the outermost regions of the cuticle, which ontogenically correspond to its earlier manifestations (Riederer and Schönherr, 1988; Heide-Jørgensen, 1991; Jeffree, 1996), may have a higher degree of packaging as compared to the regions localized below, hence having a lower degree of reactant infiltration (and labeling of specific constituents, as described below). In addition, due to the low specificity of some commonly used stains (Hayat, 1993; Soukup, 2014), cuticle histochemical analyses should also be interpreted with caution.

More specific immuno-chemical studies have been applied for identifying the presence and location of cuticle constituents such as polysaccharides (Tenberge, 1992; Guzmán et al., 2014c) and cutin (Domínguez et al., 2010; Kwiatkowska et al., 2014). However, while immuno-gold particles indicate the presence of such cuticle chemical constituents, no specific features or degree of electron-density can be observed in the labeled zones (e.g., Guzmán et al., 2014c), the results being of qualitative rather than structural value. It must be also considered that the absence of these particles in a labeled cuticle does not directly imply the absence of the target constituent, which may be masked by other cuticle chemical compounds, as derived from the results by Guzmán et al. (2014c versus 2014a).

Various attempts have been made to infer the chemical composition of alternating electron-lucent and electron-dense lamellae observed in the outer region of some plant cuticles (see Jeffree, 2006) and further trials will be required to examine the nature of such lamellae. Working with cuticles isolated from two different eucalypt species (*Eucalyptus globulus* and *E. camaldulensis*), Guzmán et al. (2014a) observed the disappearance of the lamellate structure occurring underneath the epicuticular wax layer after organic-solvent extraction, while the same extraction procedure did not affect the appearance of the lamellae found in *F. elastica* leaf cuticles (Guzmán-Delgado et al., 2016). Nevertheless, experimental evidence for the relationship between individual intracuticular wax compounds and cuticle ultra-structure has not been provided so far. Selective analyses of epi- and intra-cuticular waxes obtained by mechanical sampling followed by solvent extraction (e.g., Jetter and Schäffer, 2001) showed that cyclic compounds such as triterpenoids tend to accumulate within the intracuticular wax layer of diverse species and organs (Buschhaus and Jetter, 2011; Buschhaus et al., 2015). Trials developed with fruit cuticles of *Diospyros kaki* var. Fuyu by Tsubaki et al. (2013) indicate that triterpenoids in the cuticular matrix construct a nano-composite and provide toughness to the cuticle by functioning as nano-fillers. A similar intracuticular filler role has been proposed for flavonoids in tomato fruit by España et al. (2014).

After analyzing cutin monomer composition and the potential molecular structure adopted by this polymer in the cuticles of *Hedera helix* leaves and tomato fruit, Graça and Lamosa (2010) hypothesized that the linearity of the polymer can account for the ordered lamellae observed in *H. helix* cuticle while the branched polymer of tomato could be the basis of its reticulate structure. Nonetheless, the differences found in the potential degree of branching in the cutin of *E. globulus* and *E. camaldulensis* (having similar lamellate structure than *H. helix*, Viougeas et al., 1995) could not be assigned to a specific structural pattern, neither differences in wax composition (Guzmán et al., 2014a). By contrast, the cell wall observed in these eucalypt cuticles after successive chemical extractions had diffuse and helicoidal structural patterns in the outer and inner regions, respectively, which may lead to the different morphology of such cuticle regions. However, the possibility that the extraction processes have disrupted the orientation of the cellulose fibrils should be considered (Guzmán et al., 2014a).

It has been suggested that the presence of cutan is restricted to the cuticle region localized below the so-called ‘cuticle proper’ (Schmidt and Schönherr, 1982; Riederer and Schönherr, 1988). Recent studies (Guzmán et al., 2014a; Guzmán-Delgado et al., 2016), however, noted that the extraction of cuticle constituents is likely to be incomplete due to the presence of cutan, and that the loss of inner cuticle regions (i.e., those located closer to epidermal cell protoplasts) after cuticle de-esterification reactions also occurred in cutan-free cuticles. Therefore, the presence of cutan may further hinder the interpretation of cuticle ultra-structure in relation to composition due to the limited effect of chemical removal (Guzmán-Delgado et al., 2016).

Since the 1960’s (e.g., Leyton and Armitage, 1968; Lange, 1969) scanning electron microscopy (SEM) was used as tool for examining a great number of plant cuticles as compared with the more limited use of TEM, probably due to the easier sample preparation procedures. With some exceptions (e.g., Hill and Dilcher, 1990), SEM has been generally used for assessing morphological features of cuticle periclinal surfaces and especially of epicuticular waxes.

Several reports assessed the relationship between epicuticular wax morphology (as observed by SEM) and chemical composition (Baker, 1982; Barthlott et al., 1998; Jeffree, 2006). However, the results available are generally based on total cuticular wax composition, i.e., comprising both epi- and intra-cuticular waxes, and the contribution of specific compounds could only be inferred indirectly (Buschhaus and Jetter, 2011). Selective extractions and analyses of epicuticular wax structures can also provide a more accurate assignment of chemical compounds (Buschhaus and Jetter, 2011).

The application of electron microscopy tomography together with sample preparation by freeze substitution or high pressure freezing techniques (e.g., Seguí-Simarro et al., 2004; McIntosh et al., 2005; Austin, 2014) has not been applied to plant cuticles in intact or isolated tissues and may provide interesting structural information.

The allocation of cuticular waxes may be based on the physico-chemical properties of common cuticular components (Buschhaus and Jetter, 2011). Taking into account the polar, apolar and hydrogen (H)-bonding properties of the functional groups forming common wax types such as alkanes, alcohols, amyrins or β-diketones, Khayet and Fernández (2012) calculated the solubility parameter of various model wax compounds. Alkanes are fully apolar molecules and had the lowest total solubility parameter (i.e., the lowest surface free energy) values, whereas molecules containing oxygen in their functional groups (e.g., alcohols, acids, ketones or ester bonds) had some degree of polarity and H-bonding interactions and a higher total solubility parameter as compared to alkanes. The authors suggested that if there are no physical restrictions, compounds with a low solubility parameter (i.e., surface free energy) will tend to migrate from the plant cell wall toward the epicuticular wax layer in order to decrease the Gibbs free energy (Khayet et al., 2002). This could be an alternative and/or complementary hypothesis to explain the migration of cuticular material (e.g., waxes, cutin monomers or phenolics) toward the air-plant interface, in contrast to cuticular transpiration as a driving force (Neinhuis et al., 2001; Koch et al., 2004; Heredia-Guerrero et al., 2010).

## Building the Cuticle Puzzle: Structure of Individual Chemical Constituents

Due to the complexity in interpreting cuticular ultra-structure in relation to chemical composition, some studies focused on assessing the potential structure of isolated waxes (e.g., Jeffree et al., 1975; Matas et al., 2003; Dragota and Riederer, 2007; Dora and Wandelt, 2011), cutin monomers (e.g., Ray and Stark, 1998; Heredia-Guerrero et al., 2009; Graça and Lamosa, 2010) and polysaccharides (pectin-lipid nanoparticles, Guzman-Puyol et al., 2015). Few studies attempted to clarify the contribution of individual cuticular chemical fractions to the rheological properties of plant cuticles, e.g., waxes (Tsubaki et al., 2013), cutin (Round et al., 2000), polysaccharides (López-Casado et al., 2007) or flavonoids (Domínguez et al., 2009) but it is likely that the extraction of cuticular fractions (e.g., polysaccharide removal from cuticular membranes) may have not been complete due to the dense membrane character of isolated cuticles as observed by TEM. Trials with waxes and cutin monomers have shown that such molecules may undergo self-assembly (Domínguez and Heredia, 1999a; Casado and Heredia, 2001; Koch et al., 2009; Heredia-Guerrero et al., 2011).

### Wax Structure

Using SEM, several classifications were proposed based on epicuticular wax structure (Amelunxen et al., 1967), and also considering chemical composition (Baker, 1982; Barthlott et al., 1998; Jeffree, 2006; Koch et al., 2006a). However, the methodology commonly used to extract soluble cuticular lipids, i.e., by organ or cuticle immersion in organic solvents, does not enable to distinguish between epi- and intra-cuticular waxes. Thus, various methods for selective isolation of waxes have been developed (e.g., Buschhaus and Jetter, 2011).

The performance and re-crystallization of isolated leaf waxes from some species grown in an array of artificial substrates have been examined in several studies (e.g., Reynhardt and Riederer, 1994; Matas et al., 2003; Koch et al., 2004; Ensikat et al., 2006). Research efforts have focused on analyzing the re-crystallization mechanisms of waxes with tubular morphology extracted, for example, from *Pinus halepensis, Picea pungens, Nelumbo nucifera* or *Tropaeolum majus* (Jetter and Riederer, 1994; Matas et al., 2003; Koch et al., 2006b, 2009). Waxes are generally complex chemical mixtures and their chemical composition is decisive for the formation of e.g., a tubular or planar morphology (Jeffree, 2006; Koch et al., 2006b, 2009).

According to Jetter and Riederer (1994) the tubular and planar crystals regularly found on the same plant surfaces may be understood as the two modifications of the solid state of nonacosan-10-ol, with tubules being formed under kinetic control while thermodynamic control may lead to the planar modification. Koch et al. (2009) noted that nonacosan-10-ol and alkanediol fractions led to the formation of tubules which grew out of an underlying uniform amorphous wax film. This suggests that crystal growth is due to a self-assembly process as suggested by several authors (e.g., Casado and Heredia, 2001; Matas et al., 2003; Koch et al., 2004, 2009). Although several aspects of the mechanisms of wax formation have been elucidated through re-crystallization trials, some open questions remain such as the amount and kind of waxes necessary for the formation of amorphous versus crystallized structures, the potential effect of the cuticle matrix on wax crystallization, or the effect of environmental modifications during growth.

### Cutin and Cutan Structure

Cutin can be defined as an insoluble, aliphatic bio-macromolecule found in the outer walls of epidermal cells. It is a polyester which upon ester-breaking depolymerization gives rise to monomeric components with hydroxyl and carboxylic acid functions. The core backbone of cutin structure are C_16_ and/or C_18_ *ω*-hydroxyacids, i.e., carboxylic acids with a hydroxyl group at the opposite end of the (CH_2_)_n_ hydrocarbon chain. The C_16_ *ω*-hydroxyacids can have a saturated chain, but predominantly in many cutins, they have a secondary hydroxyl close to mid-chain. C_18_ *ω*-hydroxyacids are also often substituted at mid-chain, with two vicinal hydroxyl groups (9,10-diol) or an epoxide (9,10-epoxy). The composition of cutin varies with e.g., plant species, epidermis location or developmental stage. As a rule, the C_16_ *ω*-hydroxyacids with a mid-chain secondary hydroxyl are always a significant monomer in the composition of all cutins, but the type and relative quantity of the C_18_ *ω*-hydroxyacids are widely variable. Besides *ω*-hydroxyacids, cutin includes other building units, namely glycerol, but only in relatively minor quantities in the few cases so far analyzed (Graça et al., 2002).

The variability in monomer composition shows that at least some structural diversity must exist in cutins. Different cutin compositions and macromolecular (or supramolecular) arrangements can be behind the ultrastructural variability that can be seen by TEM in plant cuticles (Holloway, 1982). In some cases, a significant quantity of aliphatic material remains insoluble after the ester-breaking depolymerization of cutin, the residue named cutan (e.g., Guzmán-Delgado et al., 2016). This means that other types of linkages alongside ester bonds are present in cutin, or that cutan might be a structurally different bio-macromolecule.

The mechanisms by which the C_16_ and C_18_ *ω*-hydroxyacids and the other cutin building units assemble as a macromolecule are still poorly understood. The formation of cutinsomes, which are approximately 50–200 nm spherical nanoparticles resulting from the self-assembly of cutin hydroxyacid monomers in a polar environment, has been demonstrated *in vitro* (Heredia-Guerrero et al., 2008). Cutinsomes can spontaneously polymerize or cross-link cutin monomers (Heredia-Guerrero et al., 2008, 2009). They have been suggested as building units of biopolyester cutin (Heredia-Guerrero et al., 2011) and their presence has been detected *in planta* by cutin immuno-localization (Kwiatkowska et al., 2014). The interaction of pectin with polyhydroxylated fatty acids facilitating the formation of nanoparticles has been recently demonstrated and may be related to an initial step in the formation of the plant biopolyester cutin (Guzman-Puyol et al., 2015).

The study of the *in situ* intact structure of cutin, namely, its molecular structure at different organizational levels, has been hampered by its insolubility, the complexity and variability of its monomer composition, and the difficulty in separating it from other cuticle components (e.g., polysaccharides and phenolics). Typically, cutin structural studies are carried out in cutin-enriched materials, meaning isolated cuticles where waxes were extracted and polysaccharides were in part removed by enzymatic or chemical treatments. In these cutin-enriched fractions the aliphatic (acyclic or cyclic, non-aromatic compound) material can range from 50 to 80% of their mass. Cutin has been analyzed in its native (non-depolymerized) form by solid state techniques, like infra-red (Raman, FTIR) spectroscopy (Heredia-Guerrero et al., 2014) and a number of biophysical approaches. However, most of the structural information gathered so far has come from the application of nuclear magnetic resonance (NMR) techniques, summarized below.

Three types of NMR approaches have been used to study cutin as a macromolecule: solid-state NMR techniques based on magic angle spinning (MAS), high-resolution (HR-MAS) techniques applied to semi-solid cutin swelled in solvents, and high-resolution solution-state NMR of oligomer fragments, obtained by the partial depolymerization of cutin. ^13^C solid-state NMR (^13^C ssNMR), using cross-polarization (CPMAS) and direct polarization (DPMAS) techniques, together with spin relaxation studies, allowed a wealth of information regarding the structural types of carbons present, the estimation of their relative abundance and their molecular mobility or rigidity in the context of the intact cutin. Using these techniques, the pioneering work of Stark et al. (2000) in the lime fruit (*Citrus aurantifolia*) cutin, showed the presence of methylene (CH_2_)_n_ chains, primary and secondary ester groups, and carbons assignable to phenolic hydroxycinnamic moieties. Moreover, shorter relaxation times were found for the majority of the methylene carbons (ca. 60%) and for the primary head-to-tail ester carbons, showing a relatively high mobility of the latter within the macromolecular structure. In contrast, the remaining methylenes and the esters in secondary mid-chain hydroxyl positions were much more rigid, as shown by the longer relaxation times of their characteristic carbons (Zlotnikmazori and Stark, 1988; Garbow and Stark, 1990). In tomato peel cutin, the methylene (CH_2_)_n_ chains showed two signals in the ^13^C CPMAS spectra, one at 29 ppm attributed to an amorphous arrangement of gauche and anti-conformations, and another (smaller) at 33 ppm attributed to crystalline regions with the (CH_2_)_n_ chains in an all-trans conformation (Deshmukh et al., 2003).

ssNMR has the inherent disadvantage of low resolution with the broad and overlapping signals. This can be in part overcome using HRMAS techniques, if a viscous phase can be obtained from the solid cutin material, giving enhanced molecular mobility, and allowing much higher NMR resolution. HRMAS permits the use of 2D NMR techniques which can give crucial structural information, showing direct and long-range connectivities between specific carbons and hydrogens. Such an HRMAS approach was followed after swelling in deuterated DMSO cutin-enriched fractions from lime fruit (Stark et al., 2000; Fang et al., 2001), tomato peel (Deshmukh et al., 2003) and *A. americana* leaves (Deshmukh et al., 2005). Direct confirmation of the structures presumed from the ssNMR analyses was thus obtained, namely the presence of esters of primary and secondary hydroxyl groups. Besides, a number of further structural features were also found. In tomato and *A. americana* cutin, the ramification of the hydrocarbon chain in the carbon neighboring the carbonyl in ester groups (*α*-branching) was proposed, based on the chemical shift of the involved CH group (44–45 and 2.4 ppm respectively; Deshmukh et al., 2003, 2005). HRMAS analysis of the cutan residue in *A. americana* (ca. 30% of the cutin-enriched fraction) showed free carboxylic acid and hydroxyl groups, and higher degree of crystallinity in the methylene (CH_2_)_n_ chains (from a dominant peak at 32 ppm for the respective carbons), and ester groups that could be linked directly to benzene rings (Deshmukh et al., 2005).

The other approach to obtain structural information is by partially depolymerizing cutin to get oligomers still carrying the intramolecular linkages that exist in the intact macromolecule. These oligomers can eventually be isolated from the depolymerization mixtures with chromatographic techniques, allowing their unequivocal structural characterization, applying mass spectrometry and high-resolution 1D and 2D NMR techniques. Also, some specific types of intra-molecular linkages can be more or less specifically targeted, thus adding structural information. Some issues have, however, to be considered, namely the representativeness of the oligomers obtained, and the fact that some intra-molecular linkages can be more accessible than others to the partial degradative techniques employed. A number of such studies were applied to lime cutin: oligomers up to tetramers made of C_16_ *ω*-hydroxyacids linearly linked head-to-tail through esterification of their primary hydroxyls were found, after an iodotrimethylsilane hydrolysis that attacks sterically hindered esters of secondary hydroxyls (Ray et al., 1998), and after a degradative treatment made by low-temperature hydrogen fluoride (Tian et al., 2008). Alternatively, a pentamer also including mostly C_16_ *ω*-hydroxyacids, but this time esterified through the secondary hydroxyls, was obtained after an enzyme treatment that specifically cleaved the esters of primary hydroxyls (Ray and Stark, 1998). A larger set of oligomers was obtained after the partial depolymerization of tomato fruit cutin, by a mild methanolysis reaction (Graça and Lamosa, 2010). Oligomers up to heptamers, built from the C_16_ *ω*-hydroxyacids with a mid-chain secondary hydroxyl, the dominant monomer in tomato cutin were found. As proved by solution-state 2D NMR techniques, esters of both the primary (*ω*-) and secondary (mid-chain) hydroxyl groups were present, but the later were largely dominant, in a proportion of 4.5 to 1 (Graça and Lamosa, 2010).

What conclusions can be drawn from the information above regarding the *in situ* macromolecular structure of cutin within the cuticle? The cutin macromolecule certainly grows through the linear esterification of successive *ω-*hydroxyacids by their primary *ω-*hydroxyls. However, the observed extensive esterification of the mid-chain secondary hydroxyls shows a high degree of branching, which would become even denser by the local branching of the hydrocarbon chain itself. Somehow in contradiction, at least the primary ester positions and a significant part of the methylene chains, show a relatively high molecular mobility, suggesting that they are part of a not too much cross-linked network. Part of the methylene (CH_2_)_n_ chains might be part of orderly packed regions, which would answer for the molecular rigidity some of them show. These supposed crystalline-like regions were proposed to be the core of cutan (Deshmukh et al., 2005). Tentative working models were proposed for the cutin macromolecule showing a reticulate arrangement of the *ω*-hydroxyacids (Stark and Tian, 2006) or its growth in a more dendritic manner (Graça and Lamosa, 2010). Not much is known about the role of other cutin monomers, such as glycerol, or the linkages between the aliphatic cutin and phenolic moieties or the neighboring polysaccharides. A lot of research work is surely needed, using NMR and other techniques, before we can get an accurate picture of the cutin macromolecular architecture and its structural variability.

## Back to the Beginning: A Critical Examination of the Prevailing Cuticle Model

Studies developed during the last 50 years enabled a better understanding of cuticle composition, chiefly concerning cutin and waxes and, to a lesser extent, of the cuticle structure of some plant species and organs. However, as noted by Buschhaus and Jetter (2012): – ‘The contribution of various cuticle constituents to each cuticle function is currently unclear. Similarly, the contribution of the different substructures to the different roles the cuticle plays remains uncertain. Our understanding is mainly hampered by the fact that the previous investigations aimed at chemically and biologically characterizing the cuticles from different species, and then searching for structure-function correlations based on species comparisons. Nevertheless, such descriptive comparisons were necessarily confounded by the multitude of cuticle differences found between species, rather than just the one factor being assessed’ –.

Several studies used *Arabidopsis thaliana* (L) Heynh as model for cutin biosynthesis (e.g., Nawrath et al., 2013; Go et al., 2014; Fabre et al., 2016; Li et al., 2016), but its cuticle composition is atypical and not representative for most plant species (Franke et al., 2005; Pollard et al., 2008; Domínguez et al., 2011). Due to the increased availability of tomato (*Solanum lycopersicum* L.) genomic resources, this fruit is also being used as model for analyzing the mechanisms of cuticle formation (e.g., Martin and Rose, 2014; Petit et al., 2014). The commercial importance and ease in isolating the cuticle of tomato fruit (Petit et al., 2014) yields this species interesting for carrying out cuticular studies (Petit et al., 2014). However, there is evidence that tomato originated in the Andes region of South America, was domesticated in Mexico and cultivated in Europe since the 16th century, the domestication and selection processes being focused on improving fruit appearance and quality (Paran and van der Knaap, 2007). As a result of such breeding and selection efforts, an array of tomato varieties with different sizes, quality traits and colors are available in the market, many of which are susceptible to cracking (Domínguez et al., 2012). Hence, the intensive breeding and selection for quality attributes (e.g., larger sizes and improved red color) tomato fruit has undergone over the years, may limit the significance this cuticle as model for a wide range of plant organs and species, since it may rather be an example of a somehow artificial and singular cuticle.

The current situation is such that little is known about the relationship between cuticle chemical composition and structure and about the potential cross-links within and among cuticle constituents (Pollard et al., 2008; Guzmán et al., 2014a). Furthermore, when revising the existing literature, it can be observed a tendency toward developing sometimes detailed chemical analyses of plant cuticles (e.g., López-Casado et al., 2007; Jetter and Riederer, 2016) without considering the structure and localization of cuticle constituents which hinders the significance of the results in physiological and anatomical terms. Similarly, most molecular biology studies developed with plant cuticles (largely tomato and *Arabidopsis*) do not consider key chemical and structural features, hence limiting their overall interpretation. In summary, it can be said that there are many studies focusing on certain aspects of plant cuticles, but there is a need for an integrative approach that may help us understand their formation, structure and function.

Additionally, most researchers still understand the cuticle as a lipidic, hydrophobic layer which is independent from the epidermal cell wall underneath, a concept developed more than 150 years ago which has not been critically examined for decades. As stated in the historical overview provided above, the prevailing cuticle interpretation has been brought recently into question (Guzmán et al., 2014a,c).

The terms ‘hydrophobic’ and ‘lipophilic’ are widely used in reference to plant cuticles, while they are merely qualitative and inadequate in physico-chemical terms. Hydrophobic and hydrophilic mean that a substance has low or high affinity for water, respectively. The majority of existing molecules are formed by functional groups which provide apolarity, polarity or H-bonding interactions (Khayet and Fernández, 2012). Hence, waxes are largely apolar (strictly apolar in the case of alkanes) but will have some degree of polarity and H-bonding interactions, should they contain functional groups having oxygen (e.g., acids, alcohols or ketones; **Figure 1**). This will also hold true for cutin monomers (esterification decreases polarity and H-bonding interactions), phenolics and polysaccharides (Khayet and Fernández, 2012). Thereby, it is more accurate to discuss about the apolarity, polarity or potential H-bonding properties of plant surfaces than referring to their hydrophobic or lipophilic character. While such qualitative terms which are broadly used imply that a compound or material has significant polar or apolar components, respectively (Khayet and Fernández, 2012; Fernández and Khayet, 2015) they do definitely not reflect the physico-chemical properties of cuticle chemical constituents and should be used and interpreted with caution.

The prevailing understanding of the plant cuticle as an independent, lipidic layer, is largely based on Brongniart (1830) and von Mohl’s (1847) hypotheses, which were already controversial at that time. Such model was later supported by TEM observations of cutan-containing cuticles of *A. americana* (Wattendorff and Holloway, 1982) and *C. miniata* (Schmidt and Schönherr, 1982) which are little susceptible to chemical degradation due to the highly insoluble, and non-deesterifiable nature of cutan (Villena et al., 1999; Guzmán-Delgado et al., 2016). The methodological constraints associated with microscopic and analytical techniques as described above may additionally lead to misleading results and interpretations concerning for example, the location, quality, quantity and role of cuticle chemical constituents.

Moreover, the intimate relationship between the cell wall and the cuticle since early stages of organ ontogeny (Schieferstein and Loomis, 1959; Tenberge, 1992; Segado et al., 2016), and the permeability properties of the cuticle to water and solutes (Fernández and Eichert, 2009; Fernández and Brown, 2013) cannot be fully justified with the prevailing cuticle concept. For example, the view that the cuticle was a lipidic and hydrophobic layer, is not easily reconciled with the rates of permeability of water and electrolytes (Schreiber and Schönherr, 2009), and the existence of ‘aqueous-’ or ‘polar pores’ has been hypothesized (e.g., Schönherr, 1976, 2000, 2006). Concerning the mechanisms of water permeability, cuticle hydration capacity (Chamel et al., 1991; Luque et al., 1995a,b; Domínguez and Heredia, 1999b) theoretically reflects the occurrence of polar domains in the cuticle. These domains have been mainly ascribed to cutin (Schönherr and Huber, 1977; Becker et al., 1986), phenolic compounds (Luque et al., 1995a) or polysaccharides (Chamel et al., 1991; Domínguez and Heredia, 1999b). The supposed localization of polysaccharides only in inner cuticle regions led to the suggestion that ‘polar pores’ cross the cuticle to explain the transport of polar substances and electrolytes through the cuticle (Hull, 1970; Schönherr, 1976, 2000; Schönherr and Schreiber, 2004; Schreiber, 2005; Niemann et al., 2013). However, the existence of such ‘pores’ has not been demonstrated by microscopic means so far and the occurrence of pores as such seems unlikely given the ultrastructure of the cuticle as observed by TEM (**Figure 3** and see TEM micrograph compilation by Jeffree, 2006) of many plant cuticles reported so far (Fernández and Eichert, 2009).

In addition, the drastic reduction of cuticle water absorption capacity after polysaccharide extraction (Chamel et al., 1991; Reina et al., 2001; Domínguez et al., 2011) or the relatively low abundance of polysaccharides in the cuticle as compared to other chemical fractions (Schreiber and Schönherr, 2009), led us to hypothesize that both the amount and role of polysaccharides may be underestimated (Guzmán et al., 2014a,c), as previously suggested in the review by Domínguez et al. (2011).

## A Re-Interpretation of the Plant Cuticle as a Lipidized Epidermal Cell Wall Region

The intimate linkage between polysaccharides and lipid cuticle constituents was suggested during the second half of the 19th century and at the beginning of the 20th century (e.g., Fremy et Terreil, 1868; Cross and Bevan, 1916). The lipidized cell wall nature of the cuticle has been noted by Yeats and Rose (2013), and was structurally and chemically observed for the leaf cuticle of two eucalypt species by Guzmán et al. (2014a). The cell wall structure of these eucalypt cuticles after chemical extractions differed between the outer and inner cuticle regions of both species, with diffuse and helicoidal cellulose patterns associated with the so-called ‘cuticle proper’ and the ‘cuticular layer,’ respectively (Guzmán et al., 2014a). The presence of cellulose and pectins along cuticle transversal sections, i.e., from the innermost cuticle surface up to the epicuticular wax layer, was also demonstrated for the leaf cuticles of *E. globulus*, pear and *Populus* x *canescens* by enzymatic-gold labeling (Guzmán et al., 2014c).

We hence suggest a re-interpretation of the cuticle as a lipidic region (or regions) of the cell wall as depicted in **Figure 3B**, versus the cuticle understood as free of polysaccharides (e.g., von Mohl, 1847) or having minor (or more significant) amounts of polysaccharides stemming from the subjacent epidermal cell wall (e.g., Jeffree, 2006; Domínguez et al., 2011), as reflected in **Figure 2A**. Additionally, the concept of the cuticle as a cutinized cell wall as suggested by some authors (e.g. Domínguez et al., 2011, 2015; Segado et al., 2016) seems too narrow given the potential significance of intracuticular waxes as shown in studies developed with various species and organs (e.g., Buschhaus and Jetter, 2011, 2012; Tsubaki et al., 2013) and the potential overestimation of cutin content at the expense of other chemical fractions (Guzmán et al., 2014a).

The interpretation suggested in **Figure 2B** implies that the epidermal cell wall provides the framework for the cuticle, which may contain waxes, cutin, or cutan, phenolics and further common cell wall components (e.g., mineral elements). We would emphasize the potential within this model for modifications in relation to e.g., different species, growing conditions or organs. By TEM the cuticle of an organ is often observed as a gray to whitish layer of approximately constant thickness (see pear and poplar leaf cuticles as an example, **Figures 3A,B**), but deviations from this pattern may be found such as e.g., a thicker epicuticular wax layer (e.g., wheat leaf cuticle on **Figure 3D**) or an irregular cuticle (e.g., Magellan’s beech leaf cuticle, **Figure 3C**).

Thus, the use of the terms ‘cuticle’ (or ‘cuticle proper’) and ‘cuticular layer’ (e.g., Jeffree, 2006; Nawrath et al., 2013; Serrano et al., 2014), which were introduced to distinguish the cuticle from the underlying cell wall (with cellulose being absent or present, respectively, von Mohl, 1847) may be misleading and fails to reflect the actual chemical and structural nature of the cuticle. Consequently, we understand that it is more suitable to simply refer to the epicuticular waxes, cuticle and subjacent epidermal cell wall regions. Epicuticular waxes can be considered a layer that is located over the cuticle and may contain minor phenolic amounts. As proposed in **Figure 2B**, we interpret the cuticle as an epidermal cell wall region that contains significant lipid proportions (e.g., cutin, cutan, waxes or phenolics) in addition to typical cell wall components, in contrast to the subjacent cell wall regions which are largely made of polysaccharides (i.e., cellulose, pectins and hemicelluloses). This also implies a potentially major role for polysaccharides as structural framework for the cuticle, a role that has been often assigned to cutin (e.g., Kolattukudy, 1970, 2005; Nawrath, 2002).

Cuticular transversal sections may potentially have different regions and, in case there are two, they could be differentiated by calling them e.g., ‘outer region’ and ‘inner region’ (Guzmán-Delgado et al., 2016). Parts with different patterns are sometimes clearly distinguishable in the cuticle of some species and organs. However, some cuticles do not show this layout and hence a general cuticle structure model which may reflect the nature of different e.g., species cannot be provided nowadays (see **Figure 3** as an example; with emphasis on Magellan’s beech). The structural and chemical nature of the cuticle from different organs and species can be expected to affect, for example, the bi-directional transport of matter (e.g., gasses or liquids) between plant surfaces and the surrounding environment, the mechanical properties of plant surfaces or their performance under biotic and abiotic stress factors.

In summary, for improving the state of knowledge of cuticle structure, chemical composition and function, further integrative studies with different species, organs, under variable environmental conditions or using isolated cuticular chemical fractions should be developed. The majority of the plant cuticle investigations performed to date have been focused on chemical, structural, ecophysiological or molecular biology aspects, and there is a need for integrating such knowledge as prerequisite for improving our understanding of cuticle formation, structure and function.

New findings on cell wall composition, structure, development and response to environmental stress (e.g., Xia et al., 2014; Endler et al., 2015; Bidhendi and Geitmann, 2016; Cosgrove, 2016; Sorek and Turner, 2016; Wang et al., 2016) should be considered since they may be enlightening for interpreting cuticle development, structure and composition in relation to different developmental stages, organs or growing conditions.

For the proper interpretation of cuticle structure, composition and barrier characteristics, the polarity, apolarity and hydrogen-bonding interactions of cuticle chemical constituents must be taken into consideration. Furthermore, efforts should be made to shed light on, among other factors, the physico-chemical role, location and chemical binding (if) and/or self-assembly mechanisms between cutin monomers, polysaccharides, waxes and phenolics, also in relation to epidermal cell wall development as affected by potential biotic and abiotic stimuli.

## Author Contributions

The structure and general scope of the review was designed by VF, PG-D, and LG. All authors contributed to writing, revising and approving the final version this manuscript.

## Conflict of Interest Statement

The authors declare that the research was conducted in the absence of any commercial or financial relationships that could be construed as a potential conflict of interest.
